# Reply to: Letter regarding manuscript: KSST-D-11-00615R3: ‘A randomised controlled trial for the effectiveness of intra-articular Ropivacaine and Bupivacaine on pain after knee arthroscopy: the DUPRA (DUtch Pain Relief after Arthroscopy)-trial’

**DOI:** 10.1007/s00167-012-1967-9

**Published:** 2012-03-28

**Authors:** M. M. Campo, G. M. M. J. Kerkhoffs

**Affiliations:** 1 Department of Orthopedics and Traumatology, Tergooi Hospitals, van Riebeeckweg 212, 1213XZ Hilversum, The Netherlands; 2 Department of Orthopedics and Traumatology, Academic Medical Centre Amsterdam, Meibergdreef 9, 1105 AZ Amsterdam, The Netherlands; 3 Department of Orthopedic Surgery, Academic Medical Center, Meibergdreef 9, 1105 AZ Amsterdam, The Netherlands

Dear Sirs,

Thank you for your interest in our manuscript. With pleasure, we took time to read your comments. In general, we would like to state that it is obvious that high level of evidence research costs time for design of a study, time for the study to be executed and results to be analysed and subsequently for the results to be published. In this time period, the evidence- and eminence-based opinion based on latest literature can change. This, however, does not implicate that results from randomised clinical trials with negative outcome should not be shared with the research community.

Allow us to comment that you quote the study performed by Farkas et al. [3]. This study is methodologically very solid. However, it remains an in vitro study with partly monolayer cultures tested, and partly with osteochondral samples, where both had long times of exposure to the agents, and in the osteochondral sample group, only the superficial layer was affected. There are also in vivo studies to quote when commenting that intra-articular use of Bupivacaine seems not safe.

The DUPRA trial was primarily designed to come up with level 1 evidence regarding the effectiveness of single dose intra-articular Ropivacaine and Bupivacaine after arthroscopy. During the design of the DUPRA trial, the first in vivo studies on the safety aspects of intra-articular injection of Bupivacaine were not yet published, and therefore the DUPRA trial was without concern regarding safety approved by the medical ethical committee of the University of Utrecht. During inclusion, publications on the safety of intra-articular Bupivacaine and Ropivacaine came out; the first being an in vitro study by Chu et al. [2]. Other in vitro studies followed and raised general concern. The first well-designed in vivo studies were published in 2010 [1]. At this time, inclusion of our study was completed and data analysis was started. Study results were then presented at congress meetings as well as a manuscript was written and prepared for publication in the KSSTA journal.

Although the aforementioned information gives a clear picture on how it works in the field of research and publication, we felt that the information from the DUPRA trial was still very much worth sharing with other researchers in the field considering that our outcomes provide an extra definitive argument to stop with the daily use of intra-articular injection of Bupivacaine or Ropivacaine after arthroscopy.

Allow us to explain. In daily practice, the discussion on safety aspects remained viable for the use of single-shot, low doses of an intra-articular injection with Bupivacaine or Ropivacaine, simply because of the fact that numerous colleagues were not convinced of the chondrotoxic effect of low-dose single-shot intra-articular injections—Bupivacaine and Ropivacaine have time- and dose-dependent chondrotoxicity [3–5]—and were convinced advocates of the beneficial effect on pain reduction of these aforementioned low doses.

The DUPRA trial failed to demonstrate clinically relevant beneficial effects of both Bupivacaine and Ropivacaine and therefore ends the discussion. Hence, there is no need to argue on the details of the safety aspects of low-dose single-shot intra-articular injections at all anymore, since there is no clinically relevant beneficial aspect to be expected.
